# Supplementary figures and images for: Arbidol inhibits human esophageal squamous cell carcinoma growth in vitro and in vivo through suppressing ataxia telangiectasia and Rad3-related protein kinase
Source: eLife. 2022 Sep 9;11:e73953. doi: 10.7554/eLife.73953 (PMC9512399; doi:10.7554/eLife.73953)

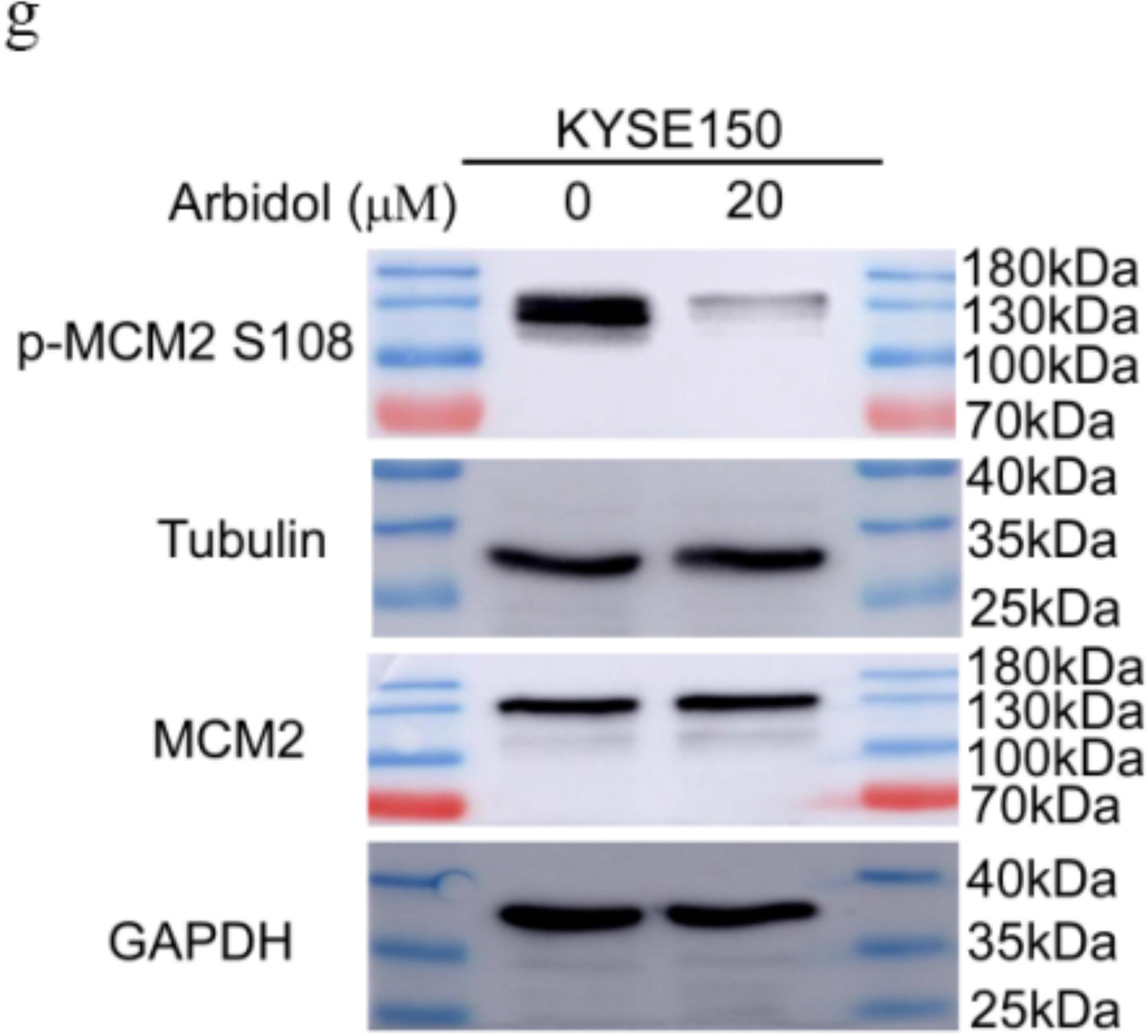

Supplement: Figure 2—source data 1. [file elife-73953-fig2-data1.tif]

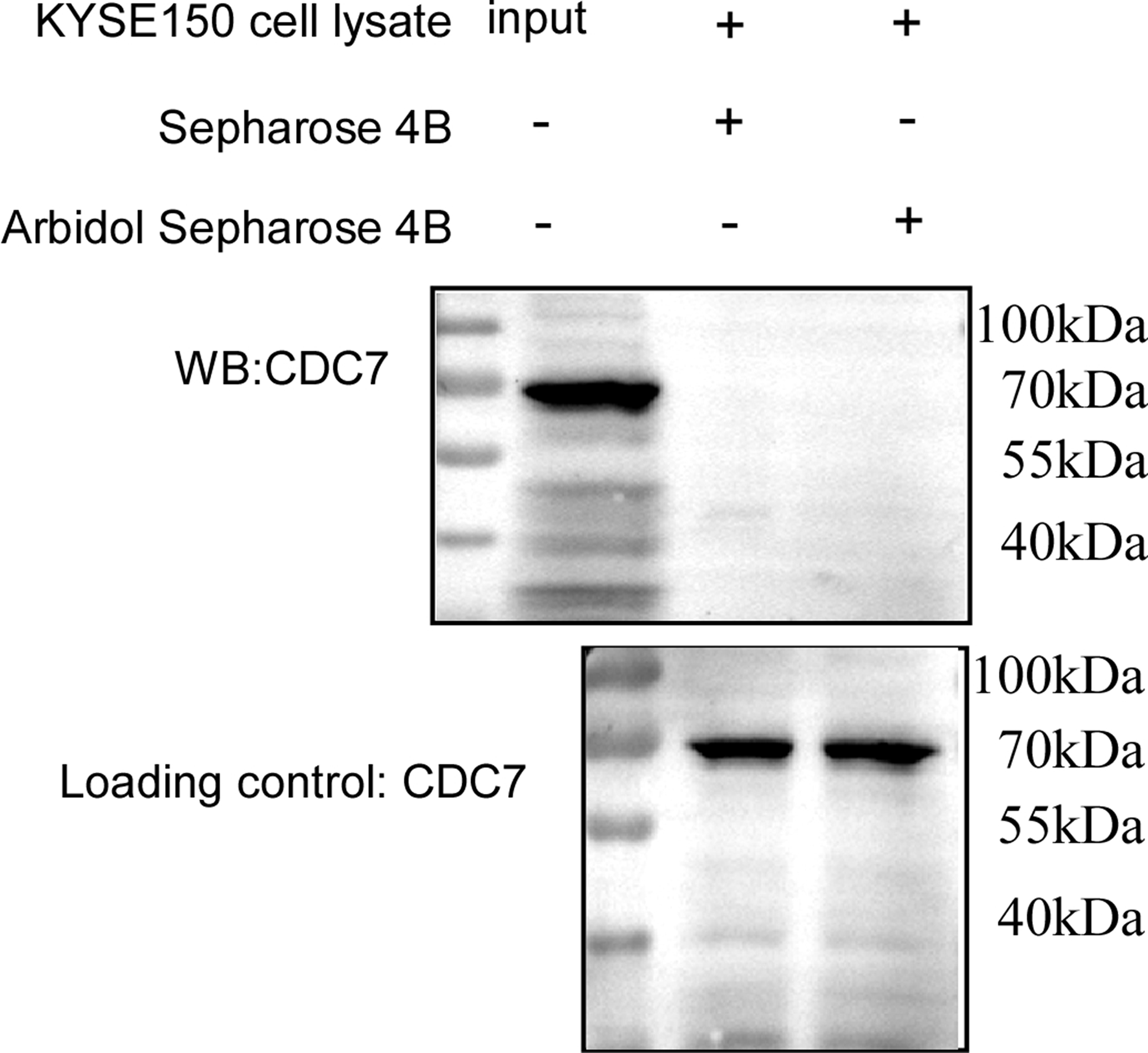

Supplement: Figure 2—figure supplement 1—source data 1. [file elife-73953-fig2-figsupp1-data1.tif]

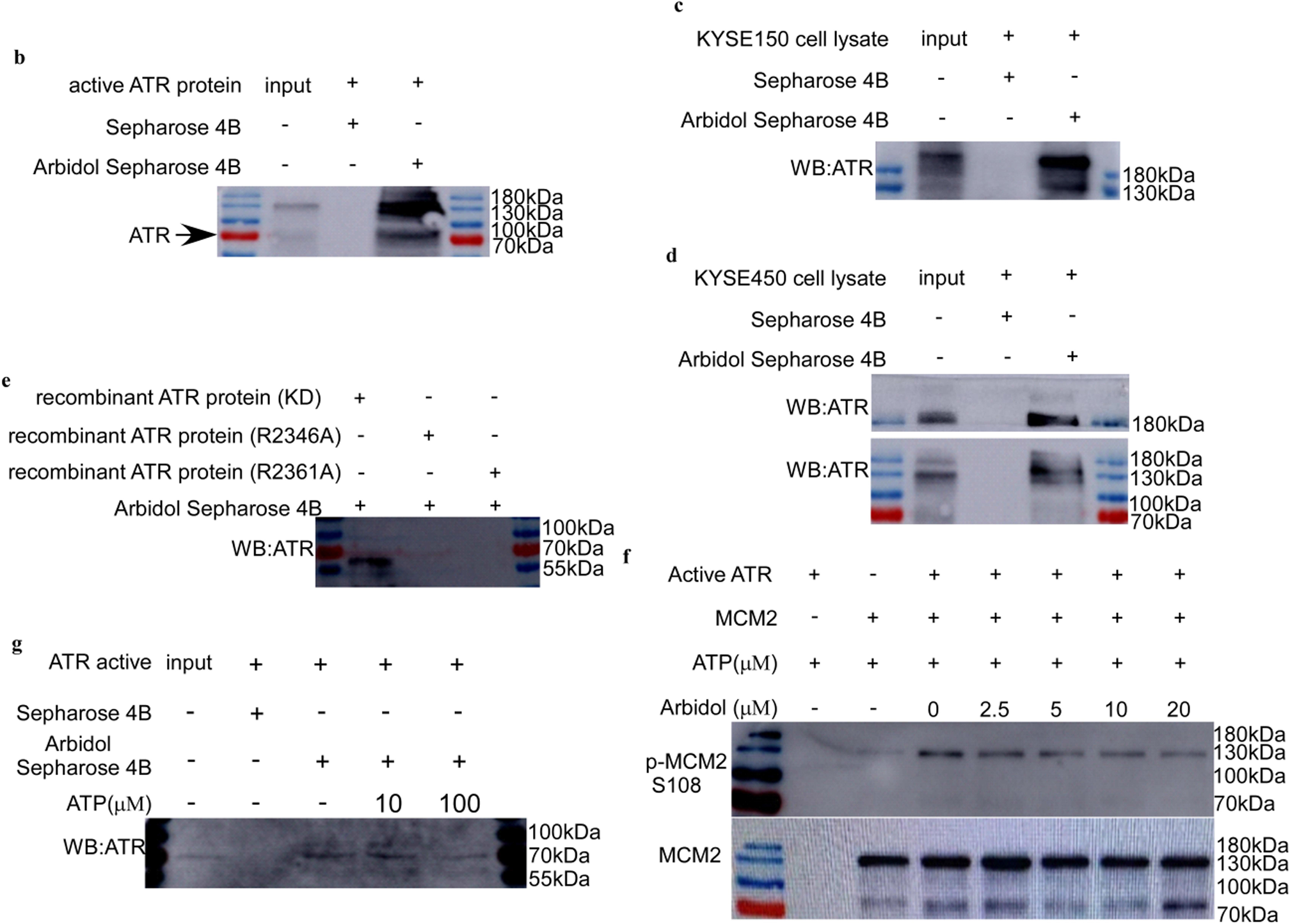

Supplement: Figure 3—source data 1. [file elife-73953-fig3-data1.tif]

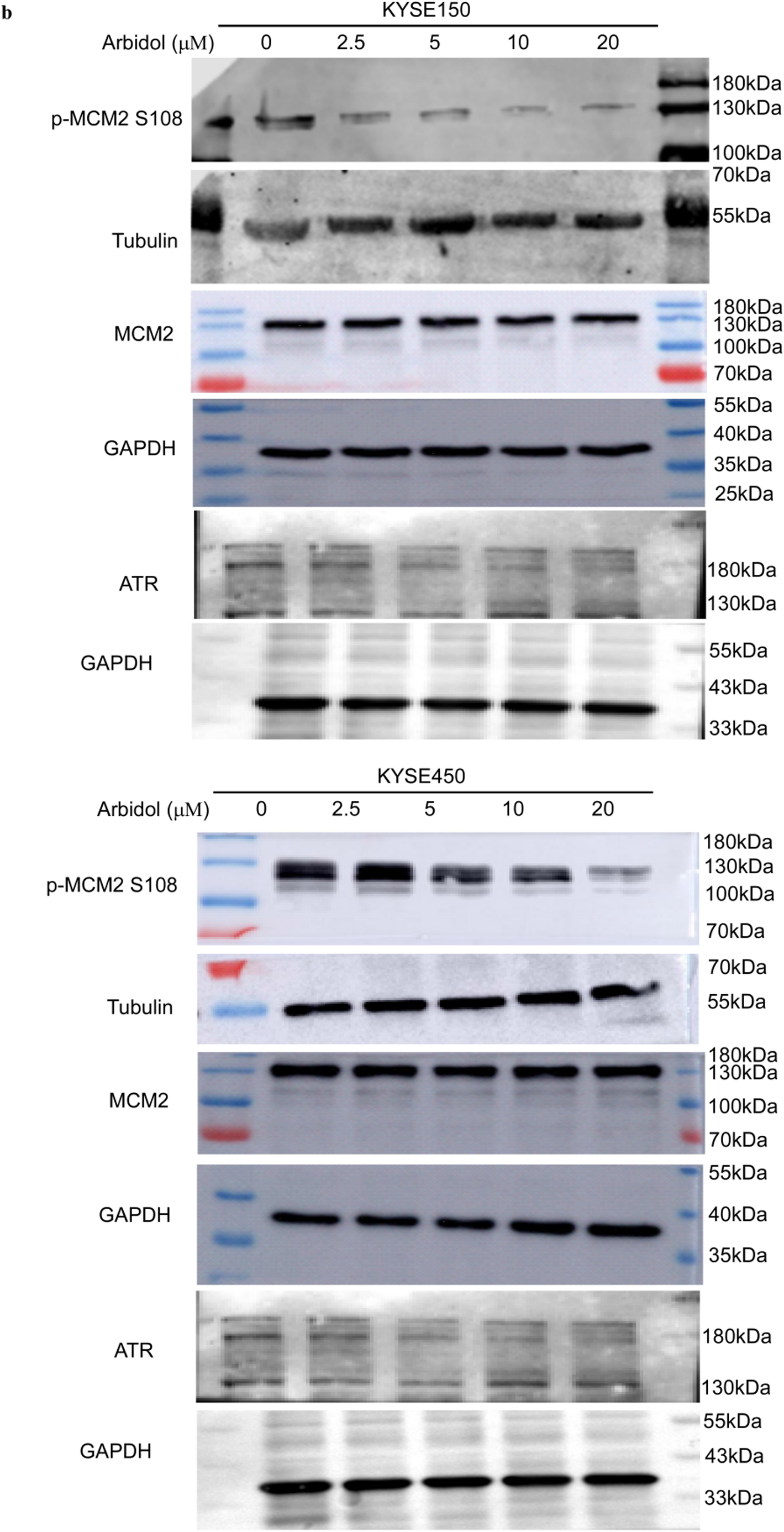

Supplement: Figure 4—source data 1. [file elife-73953-fig4-data1.tif]

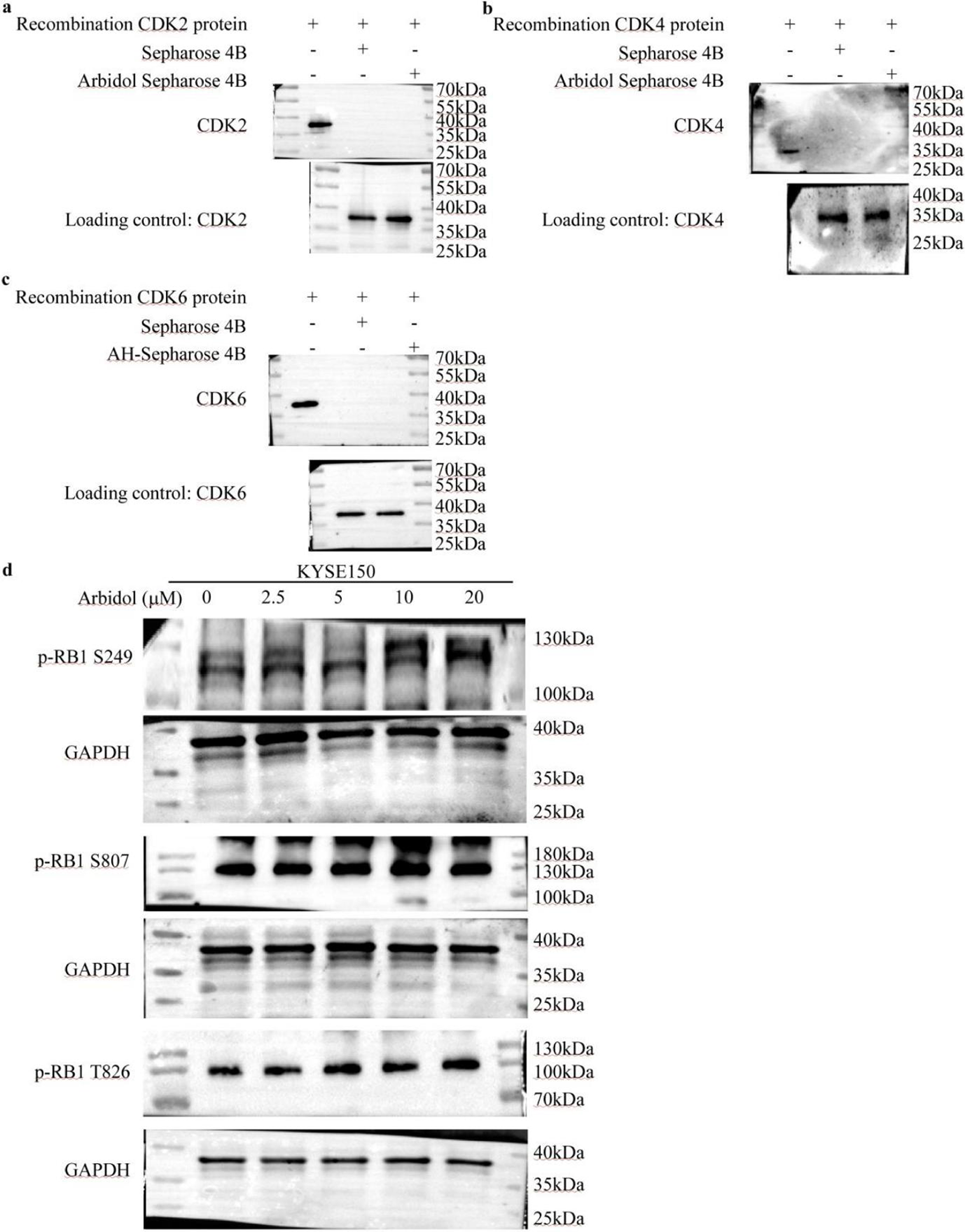

Supplement: Figure 4—figure supplement 2—source data 1. [file elife-73953-fig4-figsupp2-data1.tif]

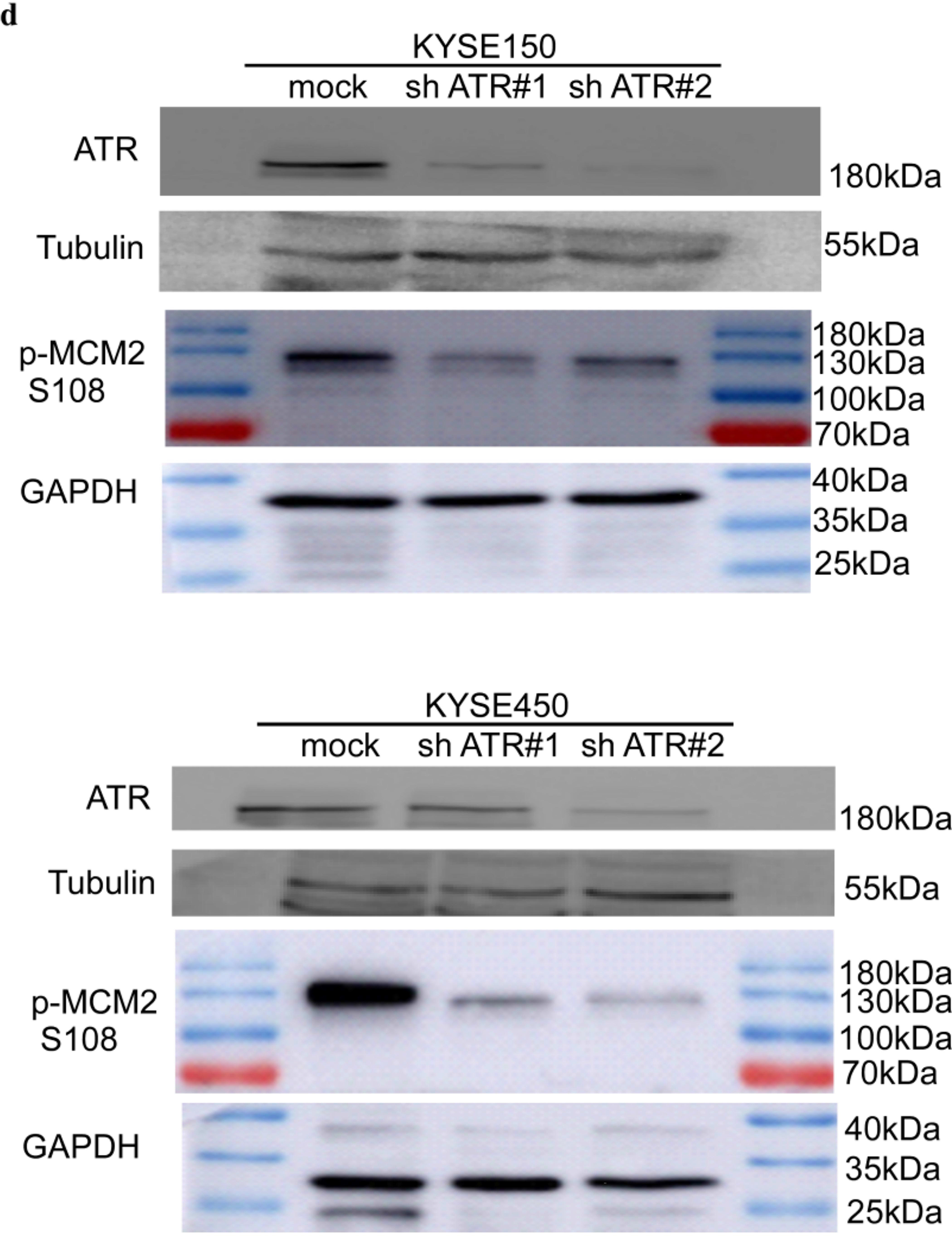

Supplement: Figure 5—source data 1. [file elife-73953-fig5-data1.tif]
